# Severe Generalized Tetanus in a Chimpanzee (*Pan troglodytes*) Under Human Care: A Case Report from the Republic of Congo

**DOI:** 10.3390/vetsci13010013

**Published:** 2025-12-22

**Authors:** Manuel Fuertes-Recuero, Juan A. De Pablo-Moreno, Luis Revuelta, Debby Cox, John Debenham, Pablo Morón-Elorza, Javier M. De Pablo-Moreno, Rebeca Atencia

**Affiliations:** 1 Department of Physiology, College of Veterinary Medicine, Complutense University of Madrid, Avda. Puerta de Hierro s/n, 28040 Madrid, Spain; manufuer@ucm.es (M.F.-R.); lrevuelt@ucm.es (L.R.); 2 Veterinary Teaching Hospital, Complutense University of Madrid, Avda. Puerta de Hierro s/n, 28040 Madrid, Spain; 3 Department of Veterinary Sciences, School of Biomedical and Health Sciences, Universidad Europea de Madrid, 28670 Villaviciosa de Odón, Spain; 4 Jane Goodall Institute Congo, Pointe-Noire, Congorebecatencia@hotmail.com (R.A.); 5 Norwegian School of Veterinary Science, Norwegian University of Life Sciences, Postbox 8146 Dep., 0033 Oslo, Norway; 6 Department of Pharmacology and Toxicology, Complutense University of Madrid, Avda. Puerta de Hierro s/n, 28040 Madrid, Spain; p-moron@hotmail.com; 7 Fundación Oceanogràfic de la Comunitat Valenciana, 46013 Valencia, Spain; 8 VISAVET Health Surveillance Centre, Complutense University of Madrid, 28040 Madrid, Spain

**Keywords:** non-human primates, great apes, wildlife rehabilitation, tetanus vaccination, wound debridement, wire-snare injury, antitoxin

## Abstract

Tetanus is a life-threatening neurological disease affecting vertebrate species, including primates. Here, we present a case of severe generalized tetanus in a juvenile male chimpanzee (*Pan troglodytes*) that was rescued from the illegal wildlife trade and admitted to a rehabilitation center in the Republic of Congo. Upon arrival, the chimpanzee presented with deep, contaminated constrictive wounds, trismus, generalized rigidity, and stimulus-induced tonic spasms accompanied by transient apnea, while remaining conscious. A presumptive clinical diagnosis was made, after which integrated care began immediately. This included meticulous wound debridement and irrigation, passive immunization with antitoxin, initiation of active immunization, metronidazole with adjunctive penicillin G, diazepam-based spasm control, multimodal analgesia, and low-stimulation nursing with oxygen supplementation, enteral nutrition, and temporary urinary catheterization. Aerobic wound culture yielded mixed flora, and a Gram stain of the feces showed large Gram-positive rods with terminal spores. Hematology tests revealed leucopenia with neutropenia and severe thrombocytopenia. The spasms ceased by day 5, at which point the diazepam dose was reduced and oral intake was increased. By week 8, he had made a full clinical recovery and was successfully reintegrated into his group. This case supports the use of pragmatic, sanctuary-adapted protocols and systematic vaccination.

## Simple Summary

Tetanus is a life-threatening disease that causes painful muscle stiffness and sudden spasms. We present the case of a young chimpanzee (*Pan troglodytes*) that was rescued from the illegal wildlife trade and arrived at a sanctuary with deep, dirty wounds and signs of tetanus. This included difficulty opening his mouth, a rigid posture, and spasms triggered by noise or touch, all while he remained fully conscious. The veterinary team acted immediately, cleaning and removing dead tissue from the wounds, administering an antitoxin to neutralize the toxin, starting a vaccination programme to protect him in the future, using antibiotics that work in low-oxygen environments, and controlling the spasms with calming and muscle-relaxing medication. He received gentle nursing in a quiet, dark room, assistance with breathing during spasms, careful feeding, and temporary urinary support. The spasms stopped by day five, and he had made a full recovery by eight weeks, returning to his group. This case shows that, with rapid and well-coordinated care, severe tetanus in great apes can be successfully treated, and underscores the importance of routine vaccination and proper wound care in protecting endangered wildlife.

## 1. Introduction

Non-human primates are widely used as models of human disease due to their close genomic, physiological, and immunological similarities to humans. The IUCN Red List classifies chimpanzees (*Pan troglodytes*) as endangered [1]. They face multiple human-related threats across their range, including live capture for the pet and entertainment trades, hunting for bushmeat, the use of body parts in traditional medicine, retaliatory killings linked to crop raiding, and wire-snare trapping [2,3,4]. These activities are driving population declines and pose significant conservation and public health risks by increasing the potential for zoonotic disease transmission [5]. In this context, sanctuaries and zoological institutions play an essential role in caring for rescued chimpanzees, while also supporting law enforcement efforts and broader conservation strategies [3,4,6,7,8].

Although the management of displaced chimpanzees in wildlife rescue centers is vital for their rehabilitation, many arrive in a compromised condition. Infants and juveniles are particularly vulnerable, frequently presenting with traumatic injuries, infectious diseases or chronic disorders acquired during illegal capture and transportation, or as a result of prior captivity [6,9,10,11]. One of the most lethal infectious diseases in primates is tetanus, with high case-fatality rates reported across species and in long-term colony records [12,13,14].

This disease is a life-threatening neurological disorder caused by the exotoxin tetanospasmin, which is produced by the bacterium *Clostridium tetani* [15]. This ubiquitous, spore-forming, anaerobic bacillus is found in soil and animal feces. It can also be found as a commensal within the gastrointestinal microbiota of many mammals (including humans), which explains why it is often found in fecal material. When these spores contaminate devitalized or puncture wounds and germinate to vegetative bacilli under anaerobic conditions, the vegetative organisms produce a potent neurotoxin that acts on the central nervous system and the autonomic nervous system (the sympathetic and parasympathetic nervous systems), leading to the muscular contractions characteristic of the illness [15]. Following the production of local toxins, tetanospasmin binds to peripheral nerve terminals, is internalized, and undergoes retrograde axonal transportation to the central nervous system. Neuronal binding could be effectively irreversible, so recovery depends on the formation of new synapses, which significantly slows clinical improvement [16,17,18].

In primates, infection typically follows the contamination of deep, constrictive or otherwise anaerobic wounds resulting from fights or bites [13,19,20,21]. The risk of tetanus is increased by wounds that are difficult to aerate, particularly injuries to the fingers, as well as by multiple simultaneous wounds [20]. This is consistent with the anaerobic respiratory ecology of the organism [20,22]. Postpartum and umbilical infections have also been described [12,15]. From a clinical perspective, affected primates typically exhibit the classic triad of trismus, generalized extensor rigidity and opisthotonus. They also display a pronounced startle response and stimulus-induced spasms, while remaining conscious until the later stages of the condition [10,11,17,18,19,21]. Early manifestations of tetanus often include reluctance to move or eat, progressive stiffness with adduction of the forelimbs, piloerection and risus sardonicus. Paroxysms are usually triggered by minor auditory, visual or tactile stimuli. They occur alongside continuous interictal rigidity, a pattern frequently observed in primate and human cases of the disease [12,19]. Cranial involvement often leads to dysphagia and impaired orofacial function. In severe cases, this can lead to aspiration pneumonia and acute airway obstruction. Autonomic dysfunction may accompany severe generalized disease, with tachycardia, fever, labile blood pressure, and apneic spells reported in advanced cases [12,15,17,19]. Although urinary retention and fecal incontinence are infrequently documented in non-human primates, they are biologically plausible consequences of sustained sphincter spasm and are well recognized in human tetanus [12,23]. These symptoms were first observed in toque macaques and have since been reported in other species of macaque, as well as in baboons and squirrel monkeys [12,13,19,20,21,24,25]. The prognosis for untreated or late-treated disease is severe and often fatal due to the risk of respiratory failure and cardiac arrest [12,21]. In human generalized tetanus, the incubation period from wound contamination to the first clinical signs ranges from 1 to 60 days (median around 8 days). Muscle rigidity and spasms usually peak during the second week of illness, and life-threatening complications such as laryngeal spasm with respiratory arrest and autonomic dysfunction with arrhythmias typically appear within the first 1–2 weeks in untreated or late-treated cases [23]. Outbreaks in primates left untreated or treated too late in outdoor-housed colonies have resulted in 100% mortality in squirrel monkeys and high mortality in macaques and baboons, underlining the severity of the clinical syndrome [12,14,21,25]. Epizootiology analyses from rhesus monkeys (*Macaca mulatta*) in Puerto Rico suggest that tetanus accounted for 19.5–24.7% of all deaths prior to the introduction of vaccination, with cases clustering during the mating season due to contaminated wounds from fighting [12,14].

Natural infection does not confer protective immunity, and cases of recurrence have been reported, highlighting the importance of prophylactic measures [22]. In outdoor-housed rhesus macaque populations, mass inoculation with tetanus toxoid has eliminated clinical tetanus and produced substantial collateral benefits. These include an immediate 42.2% reduction in overall mortality. Two intramuscular doses administered one year apart yielded protective antibody titers in 93.3% of sampled adults 18 years after boosting, indicating long-term, perhaps even lifelong, protection [22,26]. Accordingly, the best practice for treating at-risk primates involves meticulous wound management to increase tissue redox potential, early administration of antitoxins to neutralize unbound toxins, the use of targeted antimicrobials with anaerobic coverage, the administration of benzodiazepines to control spasms, and the implementation of systematic active immunization to prevent future disease [12,17,18,19].

This is the first reported case of tetanus in a chimpanzee (*Pan troglodytes*). This case report describes the initial clinical presentation and diagnostics of a juvenile male chimpanzee with severe generalized tetanus, as well as the successful intensive medical management. The report offers practical guidance for veterinary teams in wildlife rehabilitation settings, emphasizing the importance of rapid, coordinated care involving meticulous wound control, passive and active immunization, targeted antimicrobials, spasm control and low-stimulation care tailored to the anatomical, physiological and behavioral characteristics of great apes.

## 2. Case Description

**Figure 1:**
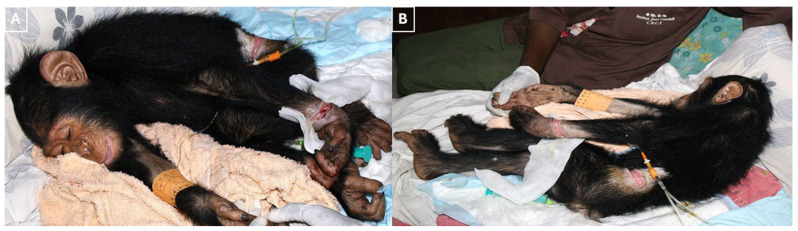
(**A**,**B**) The photographs show injuries consistent with constrictive trauma upon arrival. Deep, contaminated, circumferential lacerations are visible at the left carpus and around the wrist, accompanied by marked soft-tissue swelling and devitalized wound margins.

A six-year-old, 2.5 kg wild-born male chimpanzee (*Pan troglodytes*, a primate belonging to the great ape family Hominidae) was transferred to the Tchimpounga Chimpanzee Rehabilitation Centre in the Republic of Congo. The individual was rescued from illegal wildlife traders by the Congolese Ministry of Forest in Brazzaville, Republic of Congo. Upon arrival, the patient presented with deep, contaminated, circumferential constrictive lacerations of the left carpus and wrist (Figure 1).

**Figure 2:**
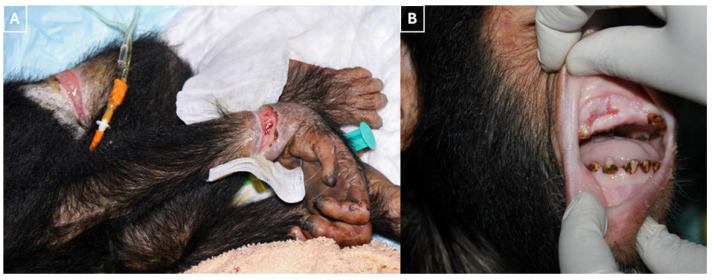
(**A**) The photograph shows the initial wound condition. The wounds were necrotic, discolored, and malodorous, with irregular, undermined edges and adherent debris. (**B**) The photograph shows dental and mucosal injury, with the absence of several incisors and a marked laceration of the buccal mucosa and adjacent soft tissues.

The wounds exhibited necrosis, discoloration, and a pronounced malodor (Figure 2A). Several incisors were missing and there was significant laceration of the oral mucosa and surrounding soft tissues (Figure 2B).

**Figure 3:**
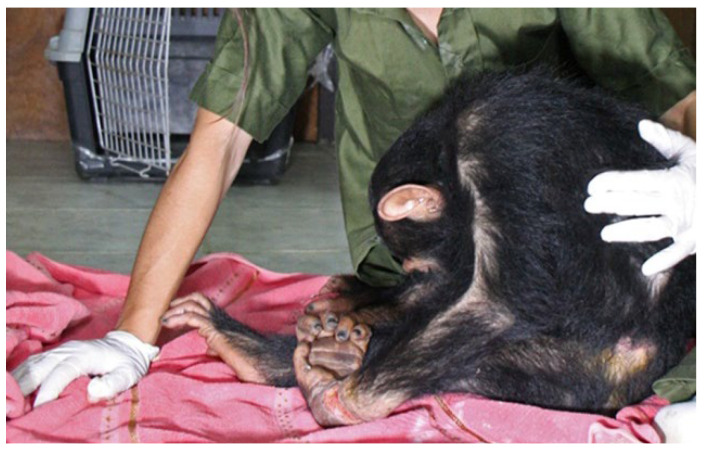
The photograph shows a tetanus-related posture at intake. The chimpanzee adopts a characteristic ‘sawhorse’ stance due to generalized spastic rigidity of the cervical, axial and appendicular muscles, which makes voluntary movement rigid and difficult.

A general examination of the chimpanzee at the wildlife rescue center revealed lethargy, anorexia, dysphagia, and mild hyperthermia (38.7 °C; physiological range from 37.0 to 38.5 °C), with preserved consciousness and generalized spastic rigidity of the axial and appendicular musculature [27,28]. The heart and respiratory rates were within the normal range (53–89 bpm and 25–40 bpm, respectively), but the chimpanzee displayed significant pain when handled. The chimpanzee adopted a ‘sawhorse’ stance due to the stiffness of its neck, abdominal, leg and arm musculature, which made body movements rigid and difficult (Figure 3). A neurological examination revealed normal mental and sensory status, alongside generalized spastic paresis. The chimpanzee experienced stimulus-triggered tonic spasms lasting from a few seconds to a minute, induced by external movements or noise. Minimal auditory or tactile stimuli triggered brief tonic episodes. During these events, the chimpanzee became apneic and exhibited opisthotonus, characterized by pronounced arching of the spine backwards with the head and heels drawn towards each other. The chimpanzee was immediately placed in a quiet, dimly lit room to minimize sensory input, and comprehensive management was initiated without delay.

**Figure 4:**
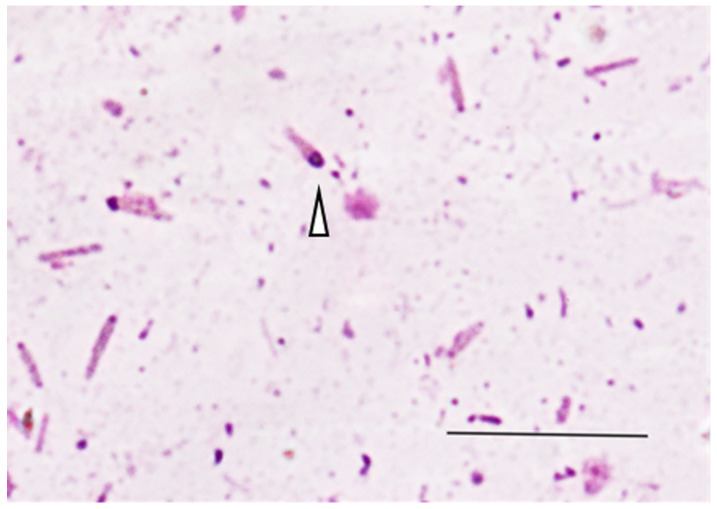
The microscope image shows a fecal sample that is compatible with clostridial morphology. The Gram stained fecal smear shows the Gram-positive bacilli exhibiting the classic racket-shaped, observing the visualization of the apical pale zone of the spore (arrowhead), all consistent with *Clostridium* spp. (Gram stain, 1000× magnification, scale: 20 μm).

Aerobic bacterial cultures were performed on swabs taken from the carpal wound in blood agar plates incubated at 37 °C in ambient air for 24–48 h and revealed a mixed flora of coliforms and coagulase-negative staphylococci. Due to site limitations and resource constraints, no anaerobic cultures were performed. Fecal Gram staining and Diff-Quik^®^ revealed large Gram-positive bacilli with polar spore formation, giving them the classic ‘racket shape’ characteristic of *Clostridium tetani* (Figure 4).

On clinical assessment, given the contaminated penetrating wounds and stimulus-induced spasms, a presumptive diagnosis of severe generalized tetanus (Ablett grade III) was made. This severity grade is characterized by marked trismus and diffuse spasticity, prolonged stimulus-evoked reflex spasms, tachypnoea with respiratory rates exceeding 40 breaths per minute, often accompanied by apneic spells, severe dysphagia, and tachycardia above 120 beats per minute, a constellation that closely matched this patient’s presentation [29]. Other differential diagnoses considered were acute electrolyte disturbance, infectious meningoencephalitis, traumatic brainstem injury, primary epileptic syndromes, and poisoning with strychnine, organophosphates, pyrethroids, or tetramine.

All wounds were promptly and meticulously debrided and irrigated with physiological saline solution. Necrotic tissue was excised from the fistula tracts to enhance tissue oxygenation and limit anaerobic proliferation.

Tetanus antitoxin (36000 IU, IM) was administered as passive immunization to neutralize circulating toxin. Active immunization with tetanus toxoid vaccine was also initiated to provide long-term protection. After 15 days, a second dose of tetanus toxoid vaccine was administered, with a booster scheduled at 12 months.

Antimicrobial therapy was initiated with metronidazole (15 mg/kg IV every 12 h; Flagyl^®^, Sanofi, Paris, France) in combination with penicillin G (20,000 U/kg IM every 12 h; bicillin^®^, Pfizer, New York, NY, USA) for 15 days.

Control of rigidity and spasms was based on benzodiazepines, with diazepam administered every 4 h (5 mg/kg IV; Covetrus, Dublin, OH, USA) and additional rescue doses during paroxysms (5 mg/kg IV; Covetrus, Dublin, OH, USA). Tramadol (2 mg/kg every 8 h PO, Cinfa, Madrid, Spain) was added for multimodal analgesia, and paracetamol (50 mg/kg every 8 h PO; Surdex, Chesterfield, MO, USA) was administered on the first day for its antipyretic effect. Oral supplementation with a multivitamin complex containing vitamins A, B, E and essential minerals (5 mL, twice daily; Alpha-Vit^®^, East Asia Laboratories, Inc, San Mateo, Rizal, Filipinas) was provided for two weeks.

Due to significant dysphagia, oral intake was limited to small, frequent volumes of thickened liquids. This approach protected the oropharynx and airway, minimized the risk of aspiration, and allowed spasms time to subside (Supplementary Figure S1).

**Figure 5:**
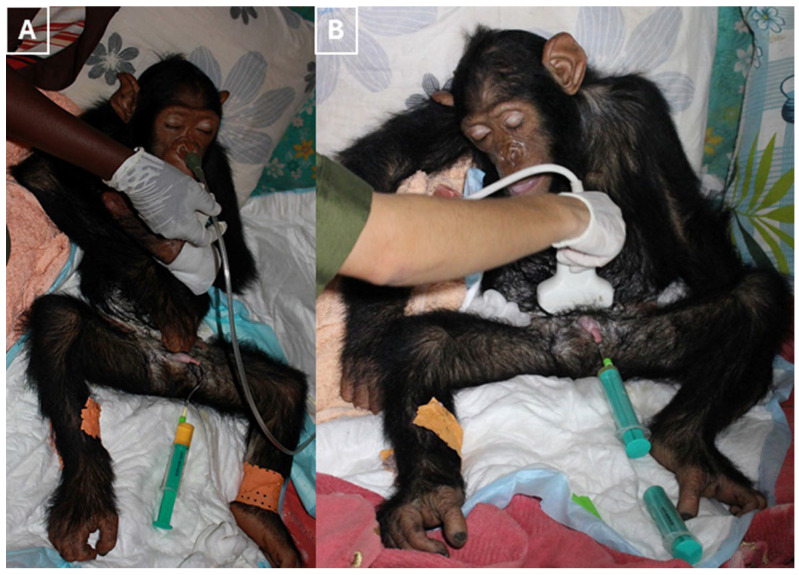
(**A**) The photograph illustrates oxygen supplementation delivered via a face mask. (**B**) The photograph depicts ultrasonography and urinary catheterization.

Episodes of apnea and transient hypoventilation associated with spasms were managed with supplemental oxygen via a face mask, and, when necessary, via a nasopharyngeal catheter, during stimulus-induced paroxysms, and did not require mechanical ventilation (Figure 5A).

Intravenous crystalloids (Ringer’s lactate at 2.5 mL/kg/h; B. Braun Medical SAS, Boulogne, France) were used for three days to ensure adequate hydration and electrolyte balance, while maintaining secured, protected venous access obtained in the cephalic vein of the right arm.

Blood samples were taken before the start of medical treatment. Hematological analysis revealed neutropenia (1.79 × 10^3^/L; normal range 1.82–7.71 10^9^/L) and leukopenia (1.42 × 10^3^/L; normal range 3.79–10.15 10^9^/L) [27,30]. Mild anemia was also detected, with hematocrit and erythrocyte counts slightly below the reference interval for the species. All other hematological parameters, including mean corpuscular volume, monocyte, eosinophil and basophil counts, hemoglobin concentration, and platelet count, were within the normal limits for *Pan troglodytes* [27]. Serum biochemistry revealed no abnormalities [27,28].

Urinary retention was observed from the day of arrival, as detected by ultrasound, which could be related to a urethral spasm. Therefore, a temporary urinary catheter was placed to decompress the bladder and monitor output. A 2 mm graduated suction catheter lubricated with sterile 2% lidocaine gel (Xilocain (Aspen Pharmacare, Durban, South Africa)) was used. Fecal retention was also observed from the day of arrival (Figure 5B). Liquid paraffin was administered orally, and a rectal enema (Microlax^®^ (Haleon, Weybridge, UK)) was given to relieve constipation. The chimpanzee was dewormed using fenbendazole (50 mg/kg orally for three days, repeated quarterly) and ivermectin (200 μg/kg subcutaneously, biannually).

During the first five days, paroxysms occurred frequently and unpredictably, with exacerbations being triggered by noise or movement. Handling was kept to a minimum and procedures were grouped together to avoid repeated stimuli. The spasms ceased at day five, allowing the diazepam dosing interval to be extended from every four hours to every six hours, and then to every eight hours the following day. The frequency of these episodes gradually reduced, with the final observed instance on day 6. From this point, the chimpanzee began to ambulate independently.

Antimicrobial treatment was completed as planned, and local wound care continued at regular intervals with sterile saline followed by topical 1% chlorhexidine (Cristalmina^®^; Supplementary Figure S2). Hemodynamic and respiratory parameters remained stable under continuous caregiver monitoring.

Recovery progressed steadily in the second week after admission, with no further paroxysms observed. From this point, benzodiazepines and tramadol were gradually tapered, with analgesia provided only as required thereafter. Dysphagia resolved alongside a progressive return to full oral intake, with hydration and caloric requirements being met entirely by mouth. The urinary catheter was removed once spontaneous micturition was sustained, and bowel function remained regular. Muscle tone continued to normalize, the startle threshold increased, and the animal became more active and engaged with its caregivers, gradually displaying species-typical behaviors such as exploration, foraging and social grooming.

In the fourth week, after rigidity and spasms had resolved, radiographic reassessment was performed under light anesthesia. The chimpanzee was anesthetized with ketamine (3 mg/kg intramuscularly) and medetomidine (0.03 mg/kg intramuscularly). Radiographs of the left wrist showed a consolidating distal both-bone forearm fracture (radius and ulna; Supplementary Figure S3). The configuration and associated soft-tissue changes were compatible with prior constrictive trauma (e.g., injury caused by a metal snare trap). No radiographic signs of osteomyelitis were observed. Upon arrival to the sanctuary, the chimpanzee was placed in quarantine and housed individually, receiving 24-h care from dedicated keepers who fed him a varied diet of fruit and vegetables and provided medical care. The chimpanzee was placed in an enclosed area with space for him and his caregiver.

**Figure 6:**
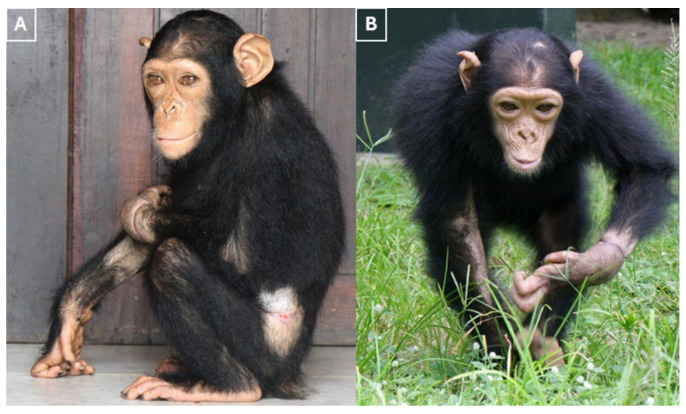
(**A**,**B**) The photographs illustrate the outcome following integrated management. The chimpanzee exhibits a normal gait and posture, displays appropriate social behavior, tolerates routine handling well, and was reintegrated into its social group.

Once the chimpanzee was clinically stable, it was moved to an enclosure next to a social group of similarly aged chimpanzees after four weeks. This allowed for protected visual and auditory contact. This was the first step in the reintegration process. Over the following days, supervised proximity was introduced without incident, eventually resulting in unrestricted group access. The chimpanzee was also vaccinated against hepatitis B, rabies and polio. By the fifth week, the chimpanzee had made a full clinical recovery. It exhibited a normal gait and posture, normal orofacial function, and displayed appropriate social interactions and tolerance of routine handling. There was no evidence of autonomic dysfunction or clinically relevant residual rigidity. Consequently, the chimpanzee was successfully integrated into a group of similarly aged chimpanzees in the fifth week, maintaining stable social behavior (Figure 6).

## 3. Discussion

This case report documents the successful medical management of severe generalized tetanus in a juvenile chimpanzee and adds detail to the very limited clinical literature on great apes, a context in which most prior reports of tetanus concern rhesus macaques, baboons, and squirrel monkeys, which show high case-fatality when treatment is delayed or inadequate [12,13,21].

In sanctuary medicine, carefully recorded case experience is valuable for the development of treatment protocols [6,11]. The clinical presentation of the chimpanzee described in our case report, preserved consciousness, persistent interictal rigidity, and stimulus-induced paroxysms accompanied by dysphagia and episodic apnea, closely matches the classical descriptions in primates and humans, and reflects the pathophysiology of tetanospasmin. This toxin is produced in devitalized anaerobic wounds, binds to peripheral nerve terminals, undergoes retrograde axonal transport and blocks inhibitory neurotransmission by severing synaptobrevin (Vesicle-Associated Membrane Protein; VAMP), thereby producing disinhibition of motor and autonomic neural circuits [12,17,18]. The functional irreversibility of neuronal binding explains both the abrupt early severity and the protracted recovery despite appropriate care [17] as it was observed in this chimpanzee.

In non-human primates, tetanus is often diagnosed clinically, as *Clostridium tetani* is difficult to culture reliably from wounds and can be present in the absence of disease. The toxin load required to cause illness is usually too low to generate detectable circulating antitoxin, meaning natural infection does not confer immunity and recurrences have been documented [22]. Although findings such as large Gram-positive ‘racket-shaped’ rods with terminal spores on fecal Gram staining or polymicrobial aerobic growth from contaminated wounds can support the diagnosis, these findings are neither sensitive nor specific and should not delay treatment [12,18,19]. In this case report, the rods were observed during fecal examination, and the acute symptoms presented by the patient prompted the initiation of early medical treatment.

Other differential diagnoses were considered upon the arrival of the patient at the sanctuary. Strychnine and other related convulsants were considered, as these also impair inhibitory neurotransmission [31]. However, the presence of deep, contaminated wounds; a subacute course with persistent interictal rigidity; and the absence of abrupt collapse or marked autonomic instability argued against this etiology. Acute electrolyte disturbances were ruled out based on blood analysis. Central inflammatory or traumatic causes were also excluded due to preserved mental status, a non-focal neurological examination and the absence of cranial nerve deficits. Primary seizure disorders were considered less likely, given that rigidity persisted between paroxysms and that episodes were consistently stimulus-evoked rather than spontaneous. Organophosphate poisoning was dismissed due to the absence of cholinergic signs such as sialorrhoea, miosis, bradycardia and diarrhea [32]. Botulism, caused by *Clostridium botulinum*, was also ruled out, as the pronounced hypertonia observed contrasts with the flaccid paralysis classically produced by botulism [32]. Therefore, in this patient, the clinical findings most strongly supported a diagnosis of generalized tetanus.

The therapeutic approach followed in this case report was based on four pillars consistent with best practices for both humans and primates in the treatment of tetanus. These pillars were: meticulous wound management to increase tissue redox potential, immediate passive immunization to neutralize unbound toxins, active immunization to provide long-lasting protection, and targeted antimicrobial therapy. This approach was established through low-stimulation nursing and spasm control [12,17,18]. Wound debridement and copious irrigation are central to limiting further toxin production from anaerobic foci [17,20]. Passive antitoxin therapy can prevent further binding of the toxin but cannot reverse the effects of the toxin that has already been internalized by nerve cells, making early administration critical [15,17]. Active immunization is required, as convalescence does not confer protection against future disease [22,26]. Metronidazole is preferred for anaerobic coverage in cases of tetanus due to its bactericidal activity in low-oxygen tissues. Penicillin is also used, despite its GABA-antagonist properties, raising concerns about potentiating spasms, particularly in severe cases [17,33,34,35]. Benzodiazepines, specifically diazepam, remain the mainstay for spasm control in primates due to their combined anticonvulsant, muscle relaxant, sedative, and anxiolytic actions via GABAergic facilitation. Evidence syntheses and veterinary reviews support their use as first-line agents [17,36]. Minimizing external triggers through quiet housing, grouping procedures, and gentle handling equally contributes to reducing paroxysms [12].

The complications observed in this case report, including dysphagia with an increased risk of aspiration, urinary retention consistent with spasm of the urethral sphincter, and refractory constipation, are mechanistically plausible consequences of tetanic co-contraction and autonomic imbalance. These complications have been documented in both the human and veterinary literature. Targeted measures, such as enteral nutrition with thickened liquids, tube feeding, temporary urinary catheterization, enemas, and rectal lavage, mitigate secondary deterioration while neural recovery proceeds [15,17]. Supplemental oxygen can compensate for transient hypoventilation during spasms, reserving mechanical ventilation for cases of refractory respiratory compromise in more advanced settings [37]. In addition to the circumferential constrictive lacerations of the left carpus, a fracture of the wrist and distal forearm was identified, which was consistent with previous constrictive trauma (e.g., a metallic wire snare used for hunting), a well-documented cause of limb injury in wild chimpanzees [2,3]. While tetanic spasms can cause fractures in humans, the pattern of injury and associated soft tissue damage in this case suggest a traumatic origin [2].

Mass tetanus-toxoid programs for outdoor-housed macaques have virtually eliminated clinical tetanus and reduced all-cause mortality by 42% in the short term. These programs have also produced durable antitoxin titers, with 93.3% of sampled adults remaining protected 18 years after boosting. This has demonstrated the feasibility and impact of systematic vaccination in primate facilities [22,26]. In sanctuaries, these data suggest the importance of combining rigorous wound management and easy access to antitoxin with scheduled active immunization and auditable record-keeping [6,9,22].

As our data are based on a single case report in a chimpanzee, they are inherently limited and cannot be generalized beyond the specific clinical and husbandry context described. Other limitations include the absence of anaerobic cultures and toxin analyses at the time of presentation, and reliance on clinical criteria rather than microbiological confirmation. Moreover, due to limited economic and technical resources for toxin-detection testing, both at the sanctuary and nationally, it was not feasible to await these results. Several investigations could not be performed because of the resource constraints at the sanctuary in the Republic of Congo [6,17]. Future studies in sanctuaries and similar low-resource settings should investigate tetX-bearing clostridia in the gut microbiome of captive chimpanzees and their potential role as a source of fecal wound contamination within a One Health framework, as previously described in human medicine [38].

In conclusion, this case report shows that severe generalized tetanus in a chimpanzee can be successfully managed in a sanctuary setting if intensive, well-coordinated medical care is provided promptly. Critical factors in achieving a favorable outcome while safeguarding welfare included early recognition, meticulous wound debridement, timely passive and active immunization, targeted antimicrobial therapy, structured spasm control, low-stimulation husbandry, and vigilant nursing. This report contributes to the small but growing body of veterinary literature on the medical management of life-threatening tetanus in great apes. Furthermore, it highlights the importance of standardized vaccination programs, the availability of antitoxin, and the sharing of detailed clinical experiences to inform future decision-making and protocol development in wildlife sanctuary medicine.

## Data Availability

The original contributions presented in this study are included in the article. Further inquiries can be directed to the corresponding author.

## Supplementary Materials

The following supporting information can be downloaded at https://www.mdpi.com/article/10.3390/vetsci13010013/s1, Figure S1: The photographs show the local wound management protocol. The wound is routinely cleansed with sterile saline, followed by the application of a topical antiseptic, at scheduled intervals throughout the acute phase; Figure S2: The photographs show the nutritional support strategy during dysphagia. This involves enteral feeding via a tube, alongside the restriction of oral intake to small, frequent volumes of thickened liquids. This protects the oropharynx and airway and reduces the risk of aspiration; Figure S3: Left forearm radiograph. Dorsoventral views show a consolidating fracture of both bones of the distal forearm (radius and ulna) at the wrist, with perilesional soft-tissue changes consistent with previous constrictive trauma. There is no radiographic evidence of osteomyelitis.

## Abbreviations

The following abbreviations are used in this manuscript:

GABA	Gamma-aminobutyric acid
VAMP	Vesicle-Associated Membrane Protein
