# Supplementary material for: Severe Generalized Tetanus in a Chimpanzee (Pan troglodytes) Under Human Care: A Case Report from the Republic of Congo
Source: Vet Sci. 2025 Dec 22;13(1):13. doi: 10.3390/vetsci13010013 (PMC12846560; doi:10.3390/vetsci13010013)
Supplement: Supplementary file 1 [file vetsci-13-00013-s001.zip › vetsci-4024941-supplementary.pdf]

Supplementary Materials

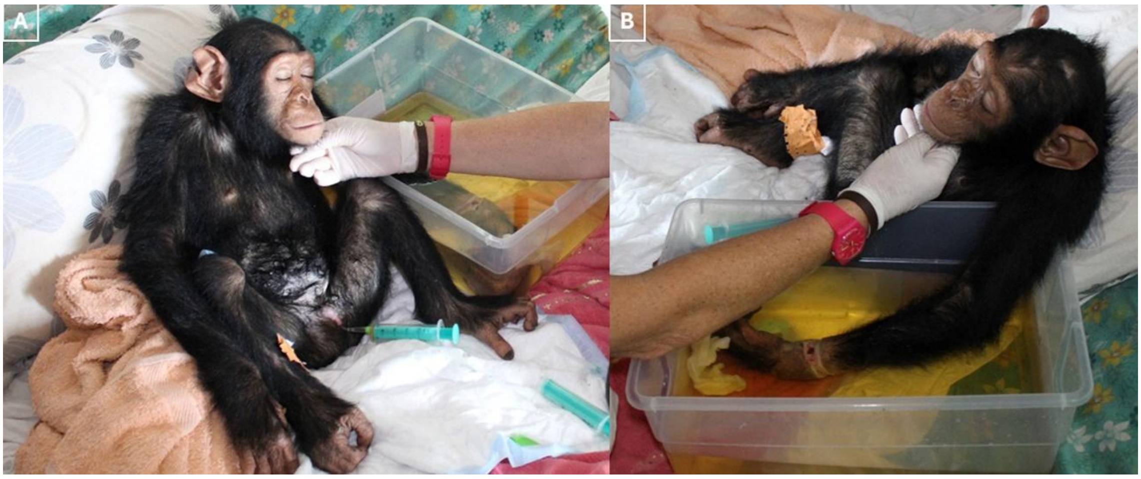

Supplementary Figure S1. **(A,B)** The photographs show the local wound management protocol. The wound is routinely cleansed with sterile saline, followed by the application of a topical antiseptic, at scheduled intervals throughout the acute phase.

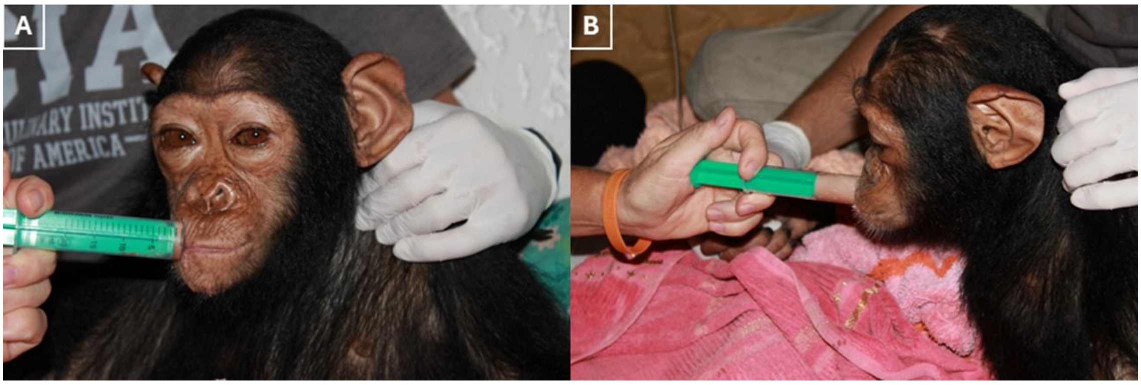

Supplementary Figure S2. **(A,B)** The photographs show the nutritional support strategy during dysphagia. This involves enteral feeding via a tube, alongside the restriction of oral intake to small, frequent volumes of thickened liquids. This protects the oropharynx and airway and reduces the risk of aspiration.

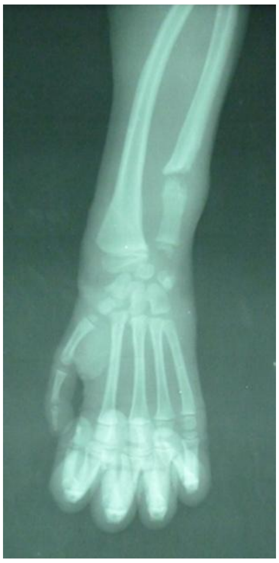

Supplementary Figure S3. Left forearm radiograph. Dorsoventral views show a consolidating fracture of both bones of the distal forearm (radius and ulna) at the wrist, with perilesional soft-tissue changes consistent with previous constrictive trauma. There is no radiographic evidence of osteomyelitis.
